# Crystal structure, DFT and Hirshfeld surface analysis of *N*-acetyl-*t*-3-methyl-*r*-2,*c*-6-di­phenyl­piperidine

**DOI:** 10.1107/S2056989022000275

**Published:** 2022-01-14

**Authors:** P. Periyannan, M. Beemarao, K . Karthik, S. Ponnuswamy, K. Ravichandran

**Affiliations:** a Department of Physics, Kandaswami Kandar’s College, Velur, Namakkal 638 182, India; b PG and Research‘ Department of Chemistry, Government Arts College (Autonomous), Coimbatore–641018., Tamil Nadu, India

**Keywords:** crystal structure, 2,6-substituted piperidine, hydrogen bonds, DFT

## Abstract

In the title compound [systematic name: 1-(3-methyl-2,6-diphenylpiperidin-1-yl)ethanone], C_20_H_23_NO, the piperidine ring adopts a distorted boat conformation, while the phenyl rings subtend a dihedral angle 65.1 (2)°. In the crystal, mol­ecules are linked by C—H⋯O hydrogen bonds into chains extending along the *b-*axis direction. The DFT/B3LYP/6–311 G(d,p) method was used to determine the HOMO–LUMO energy levels. A Hirshfeld surface analysis was conducted to verify the contributions of the different inter­molecular inter­actions, indicating that the important contributions to the crystal packing are from H⋯H (73.2%), C⋯H (18.4%) and O⋯H (8.4%) inter­actions.

## Abstract 2

In the title compound, C_20_H_23_NO, the piperidine ring adopts a distorted boat conformation, while the phenyl rings subtend a dihedral angle 65.1 (2)°. In the crystal, mol­ecules are linked by C—H⋯O hydrogen bonds into chains extending along the *b-*axis direction.

## Chemical context

The structures of a wide array of heterocyclic derivatives have been analysed for their pharma-potentiality over the past three decades (Katritzky, 2010 ▸). Among these, derivatives of the six-membered heterocyclic base piperidine have proven to be successful pharmacophores. 2,6-Substituted piperidine derivatives have been found to be useful as tranquilizers and possess a wide range of biological activities such as anti-tumor (Vinaya *et al.*, 2009 ▸), anti­viral, anti­malarial, anti­bacterial and anti­fungal activities (Aridoss *et al.*, 2009 ▸; Mobio *et al.*, 1989 ▸). These have spurred considerable awareness of the synthetic arena based on their structure, reactivity, synthesis and biological properties. We report herein the crystal structure, Hirshfeld surface analysis and DFT computational calculations of the title compound. 
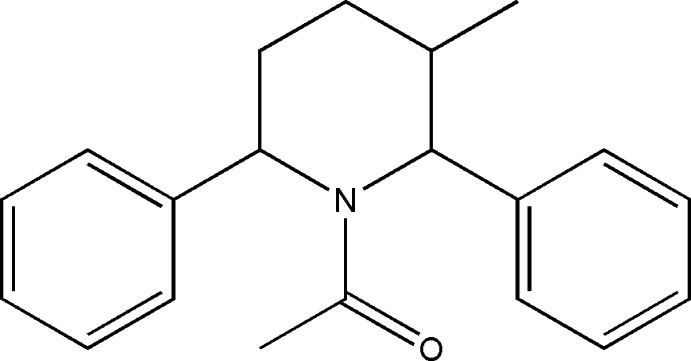


## Structural commentary

**Figure 1:**
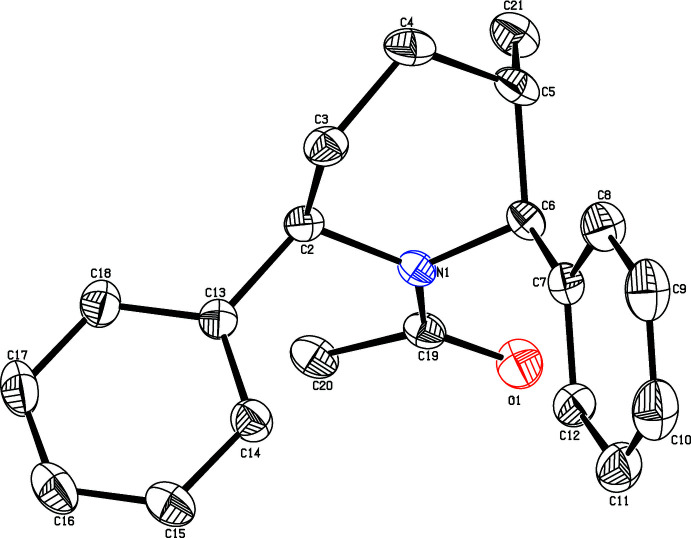
The mol­ecular structure of the title compound, showing the atomic numbering and displacement ellipsoids drawn at the 30% probability level.

The methyl-substituted piperidine title compound crystallizes in the monoclinic space group *P*2_1_. A perspective view of the mol­ecule is shown in Fig. 1 ▸. The bond lengths and angles are well within the expected limits (Roques *et al.*, 1981 ▸), and agree with values observed in related structures (Sekar *et al.*, 1990 ▸).

The piperidine ring adopts a distorted boat conformation with puckering parameters (Cremer & Pople, 1975 ▸) and asymmetry parameters (Nardelli, 1983 ▸): *q*_2_ = 0.720 (2) Å, *q*_3_ = −0.004 (3) Å, Φ(2) = 108.5 (2)°, Δ*Cs*(C3) and Δ*Cs*(C6) = 14.5 (2)°, and with maximum deviations of 0.406 (3) and 0.409 (2) Å, respectively, for atoms C3 and C6 from the best plane of the piperidine ring. The title mol­ecule contains three chiral centres *viz.*, C2, C5 and C6. The absolute configuration of the chiral centres is assigned as C2 (*R*), C5 (*S*) and C6 (*S*). The parent mol­ecule itself is chiral and the configuration cannot be changed during the substitution of acetyl group at the nitro­gen.

The sum of the bond angles (358.2°) at atom N1 of the piperidine ring is in accordance with the *sp* ^2^ hybridization state (Beddoes *et al.*, 1986 ▸). The phenyl rings at the 2 and 6-positions of the piperidine ring occupy equatorial and axial orientations. The corresponding torsion angles are C4—C3—C2—C13 = −178.8 (2)° and C4—C5—C6—C7 = −74.5 (3)°.

The piperidine ring [N1/C2–C6] makes dihedral angles of 82.0 (1) and 58.4 (1)°, respectively, with the C13–C18 and C7–C12 phenyl rings, and confirms the fact that the moieties are in axial and equatorial orientations. It is to be noted that there is a possibility of resonance between atoms N1, C19 and O1 as a result of the delocalization of the hetero π electrons of the carbonyl group, which is also confirmed by the torsion angles C2—N1—C19—O1 = 177.7 (2)° and C6—N1—C19—O1 = 13.0 (3)°.

The methyl group substituted at the 5-position of the piperidine ring is axially oriented, as confirmed by the torsion angles N1—C6—C5—C21 = −68.0 (3)° and C3—C4—C5—C21 = 112.4 (3)°, whereas the methyl group substituted at C19 is oriented equatorially with torsion angle C20—C19—N1—C6 = −166.3 (2)° and C20—C19—N1—C2 = −1.7 (3)°.

## Supra­molecular features

**Figure 2:**
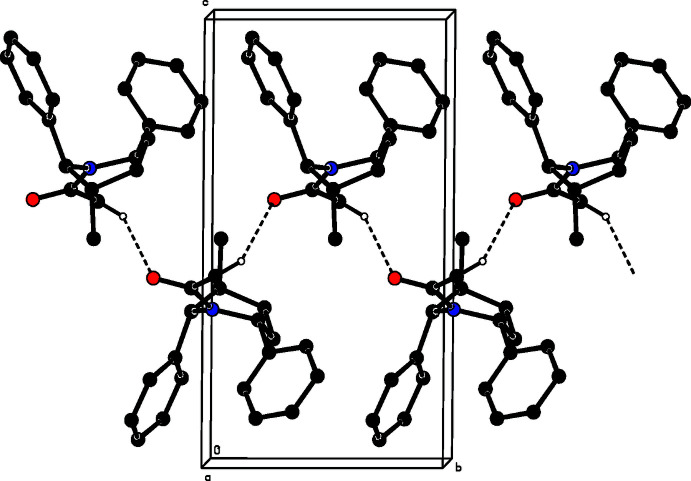
A partial view along the *b* axis of the crystal packing of the title compound, showing the formation of a mol­ecular chain by C—H⋯O inter­actions (dotted lines).

**Figure 3:**
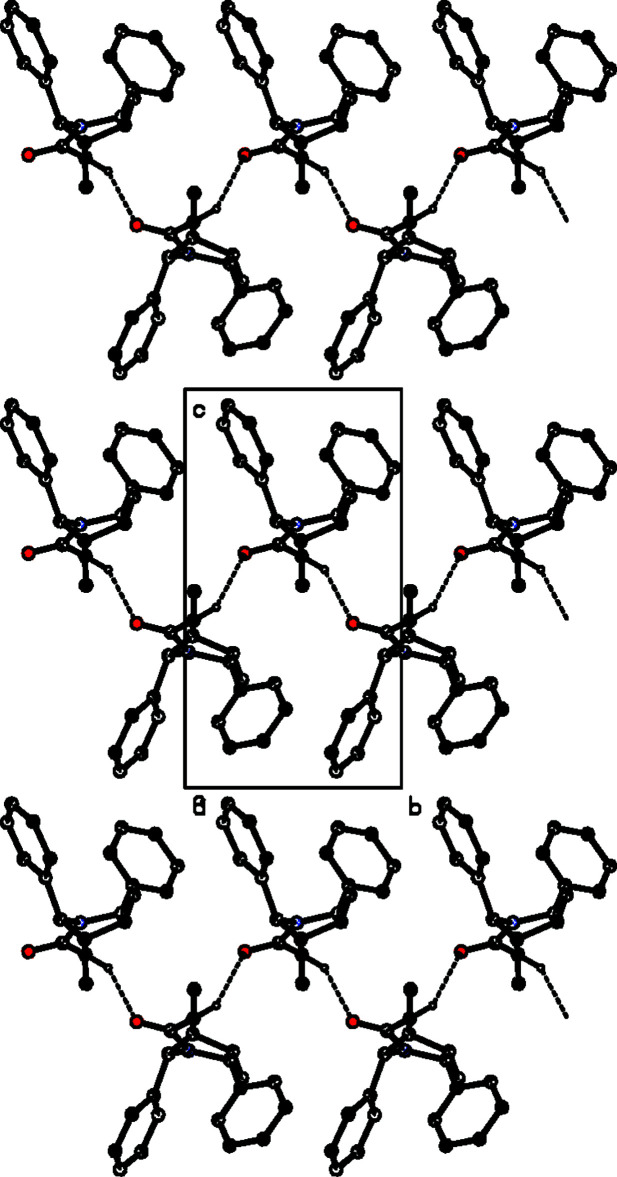
The overall crystal packing of the title compound, viewed along the *b* axis. Hydrogen bonds are shown as dashed lines, and only the H atoms involved in hydrogen bonding have been included.

The crystal packing features C—H⋯O inter­actions (Table 1 ▸). Atom C20 of the mol­ecule at (*x*, *y*, *z*) donates a proton to atom O1 of the mol­ecule at (−*x* + 1, *y* + 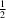 , −*z* + 1), forming a *C*4 zigzag chain (Bernstein *et al.*, 1995 ▸) running along the *b*-axis direction as shown in Fig. 2 ▸. The overall packing is shown in Fig. 3 ▸.

## Density functional theory (DFT) study

**Figure 4:**
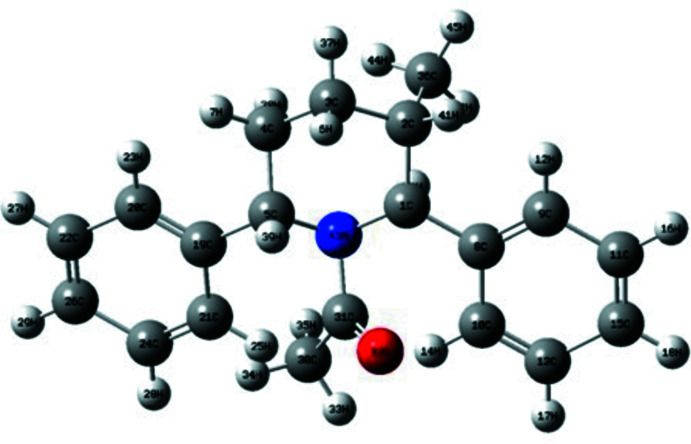
The optimized mol­ecular structure of the title compound.

**Figure 5:**
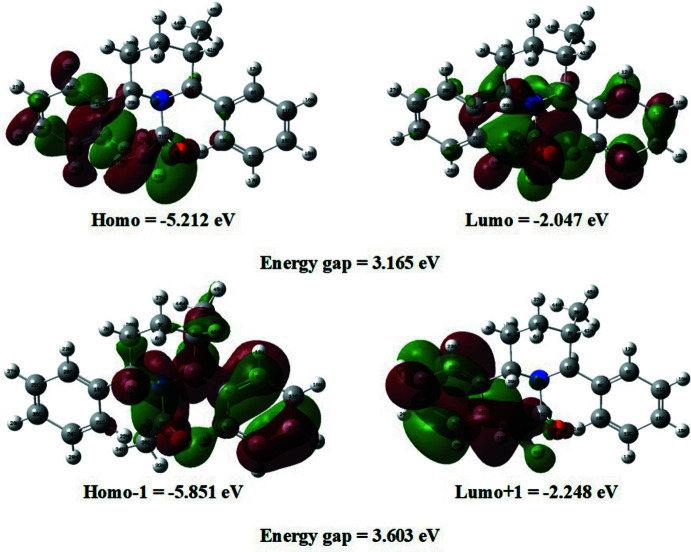
The frontier mol­ecular orbitals (FMOs) of the title compound.

The optimized mol­ecular structure and frontier mol­ecular orbitals (FMOs) (Figs. 4 ▸ and 5 ▸, respectively) were calculated using the DFT/B3LYP/6-311G(d,p) basis set implemented in the *GAUSSIAN09* program package (Frisch *et al.*, 2009 ▸). The highest occupied mol­ecular orbital (HOMO) and the lowest unoccupied mol­ecular orbital (LUMO) are called frontier mol­ecular orbitals (FMOs) as they lie at the outermost boundaries of the electrons of the mol­ecule. The electron distribution (ED) of the HOMO−1, HOMO, LUMO and LUMO+1 energy levels and the energy values are shown in Fig. 5 ▸. The positive and negative phases are represented in green and red, respectively.

The HOMO of the title mol­ecule is localized on one aromatic ring and the C=O group, while the LUMO is located over the whole mol­ecule with the exception of the CH_3_ group and some carbon and hydrogen atoms in the piperidine ring. Thus the HOMO/LUMO implies an ED transfer to the C=O group from the ring. The energy band gap (Δ*E* = *E*_HOMO_ − *E*_LUMO_) of the mol­ecule is 3.165 eV and the calculated frontier mol­ecular orbital energies, *E*_HOMO_ and *E*_LUMO_, are −5.212 and −2.047 eV, respectively. The title compound has a small frontier orbital gap, hence the mol­ecule has high chemical reactivity and low kinetic stability. The electron affinity (*A*) and ionization potential (*I*) of the mol­ecule were calculated using the DFT/B3LYP/6- 311++G(d,p) basis set. The values of the hardness (η), softness (σ), electronegativity (χ) and electrophilicity index (ω) for the title compound are given in Table 2 ▸.

## Hirshfeld surface analysis

**Figure 6:**
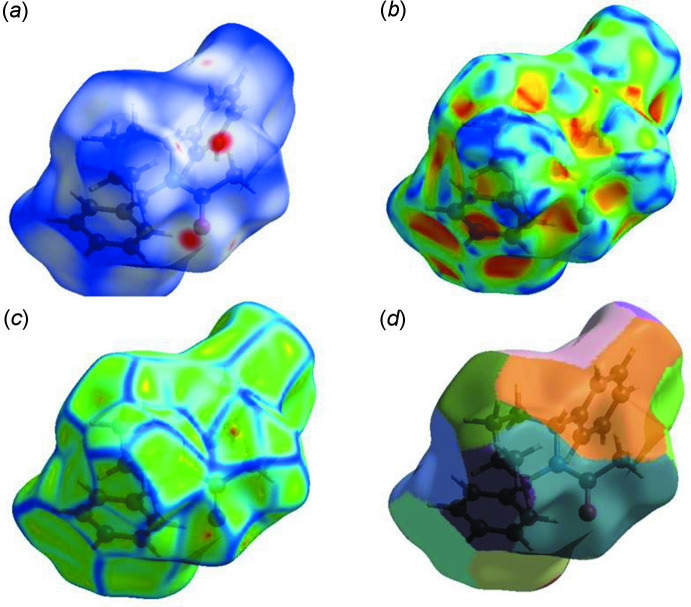
Hirshfeld surfaces mapped over (*a*) *d*_norm_, (*b*) shape-index, (*c*) curvedness and (*d*) fragment patches.

In order to visualize the inter­molecular inter­actions in the crystal of the title compound, a Hirshfeld surface (HS) analysis (Spackman & Jayatilaka, 2009 ▸) was carried out and the associated two-dimensional fingerprint plots (McKinnon *et al.*, 2007 ▸) were generated using *CrystalExplorer17* (Turner *et al.*, 2017 ▸). The Hirshfeld surface mapped over *d*_norm_ using a standard surface resolution with a fixed colour scale of −0.2 (red) to 1.3 (blue) a.u. is shown in Fig. 6 ▸ *a*. The shorter and longer contacts are indicated as red and blue spots, respectively, on the Hirshfeld surfaces, and contacts with distances approximately equal to the sum of the van der Waals radii are represented as white spots. The most important red spots on the *d*_norm_ surface represent C—H⋯O inter­actions.

The HS mapped over curvedness and shape-index, introduced by Koendrink (Koenderink, 1990 ▸; Koenderink & van Doorn, 1992 ▸), give further chemical insight into mol­ecular packing. A surface with low curvedness designates a flat region and may be indicative of π–π stacking in the crystal. A surface with high curvedness is highlighted as dark blue edges, and is indicative of the absence of π–π stacking (Fig. 6 ▸). The nearest neighbour coordination environment of a mol­ecule is identified from the colour patches on the Hirshfeld surface, depending on their closeness to adjacent mol­ecules (Mohamooda Sumaya *et al.*, 2017 ▸).

**Figure 7:**
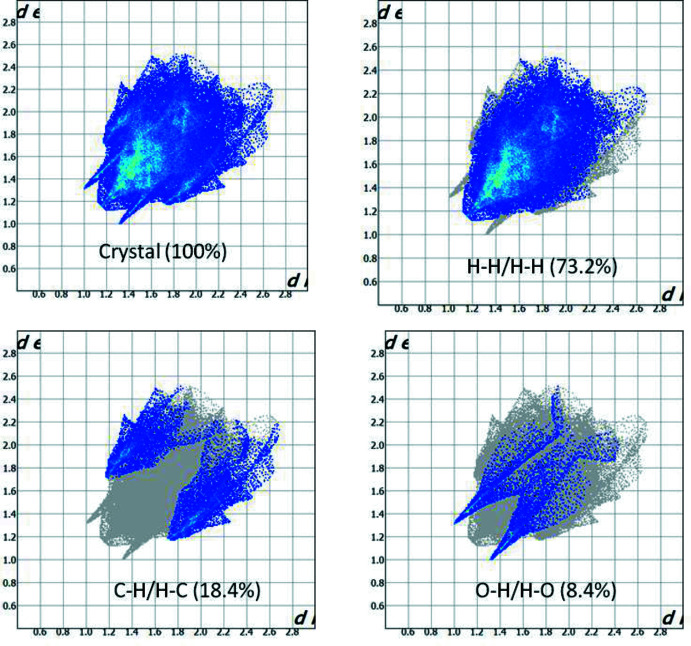
Two-dimensional fingerprint plot for the title compound showing the contributions of individual types of inter­actions (all inter­molecular contacts, H⋯H contacts, C⋯H/H⋯C contacts and O⋯H/H⋯O contacts).

The two-dimensional fingerprint plots of (*d*_i_, *d*_e_) points of all the contacts contributing to the Hirshfeld surface analysis in normal mode for all the atoms are shown in Fig. 7 ▸. The most important inter­molecular inter­actions are H⋯H contacts, contributing 73.2% to the overall crystal packing. Other inter­actions and their respective contributions are C⋯H/H⋯C (18.4%) and O⋯H/H⋯O (8.4%), respectively.

The Hirshfeld surface analysis confirms the importance of H-atom contacts in establishing the packing. The large number of H⋯H and C⋯H/H⋯C inter­actions suggest that van der Waals inter­actions and hydrogen bonding play the major roles in the crystal packing (Hathwar *et al.*, 2015 ▸).

## Database survey

A search of the Cambridge Structural Database (CSD, version 5.39, update August 2018 ▸; Groom *et al.*, 2016) for the 3-methyl-2,6-di­phenyl­piperidine skeleton yielded two hits, methyl 4-oxo-*r*-2,*c*-6-di­phenyl­piperidine-3-carboxyl­ate (BIHZEY; Sampath *et al.*, 2004 ▸) and *r*-2,*c*-6-di­phenyl­piperidine (NIKYEN; Maheshwaran *et al.*, 2013 ▸). The piperidine ring has a boat-shaped conformation in both compounds, as in the title compound. The benzene ring and the mean plane of the piperidine ring are inclined to each other by dihedral angles ranging from 19.95 to 29.16°, compared to 22.05 (6)° in the title compound.

## Synthesis and crystallization

The compound *t*-3-methyl-*r*-2,*c*-6-di­phenyl­piperidin-4-one was reduced to the corresponding piperidine using the Wolf–Kishner reduction (Ravindran & Jeyaraman, 1992 ▸). The piperidine-4-one (10 mmol) was treated with di­ethyl­ene glycol (40 ml), hydrazine hydrate (10 mmol) and KOH pellets (10 mmol) to give *t*-3-methyl-*r*-2,*c*-6-di­phenyl­piperidine. *N*-Acetyl piperidine was synthesized by the acetyl­ation of the above piperidine. To *t*-3-methyl-*r*-2,*c*-6-di­phenyl­piperidine (5 mmol) dissolved in benzene (50 ml) were added tri­ethyl­amine (20 mmol) and acetyl chloride (20 mmol) to give *N*-acetyl-*t*-3-methyl-*r*-2,*c*-6-di­phenyl­piperidine, which was crystallized by slow evaporation from a benzene and petroleum ether solution.

## Refinement

Crystal data, data collection and structure refinement details are summarized in Table 3 ▸. H atoms were positioned geometrically (N—H = 0.88–0.90 Å and C—H = 0.93–0.98 Å) and allowed to ride on their parent atoms, with *U*_iso_(H) = 1.5U_eq_(C) for methyl H and 1.2*U*_eq_(C) for other H atoms.

## Supplementary Material

Crystal structure: contains datablock(s) I. DOI: 10.1107/S2056989022000275/jy2013sup1.cif

Structure factors: contains datablock(s) I. DOI: 10.1107/S2056989022000275/jy2013Isup2.hkl

CCDC reference: 2133146

Additional supporting information:  crystallographic information; 3D view; checkCIF report

## Figures and Tables

**Table 1:** Hydrogen-bond geometry (Å, °)

*D*—H⋯*A*	*D*—H	H⋯*A*	*D*⋯*A*	*D*—H⋯*A*
C20—H20*B*⋯O1^i^	0.96	2.44	3.292 (3)	148

Symmetry code: (i) -x+1, y+{\script{1\over 2}}, -z+1.

**Table 2:** Physico-chemical properties

Parameter	Value
*E*_HOMO_ (eV)	−5.212
*E*_LUMO_ (eV)	−2.047
*E*_HOMO_ − *E*_LUMO_ energy gap (eV)	3.165
*E*_HOMO−1_ (eV)	−5.851
*E*_LUMO+1_ (eV)	−2.248
*E*_HOMO−1_ − *E*_LUMO+1_ energy gap (eV)	3.603
Ionization potential *I* (eV)	5.212
Electron affinity (*A*)	2.047
Electrophilicity Index (ω)	4.163
Chemical Potential (μ)	3.629
Electro negativity (χ)	−3.630
Hardness (η)	1.583
Softness (σ)	0.316

**Table 3:** Experimental details

Crystal data	
Chemical formula	C_20_H_23_NO
*M*_r_	293.39
Crystal system, space group	Monoclinic, *P*2_1_
Temperature (K)	296
*a*, *b*, *c* (Å)	8.3063 (4), 7.5842 (4), 13.8410 (7)
β (°)	104.174 (2)
*V* (Å^3^)	845.39 (7)
*Z*	2
Radiation type	Mo *K*α
μ (mm^−1^)	0.07
Crystal size (mm)	0.30 × 0.25 × 0.25

Data collection	
Diffractometer	Bruker SMART APEXII CCD
Absorption correction	Multi-scan (*SADABS*; Bruker, 2016 ▸)
*T*_min_, *T*_max_	0.697, 0.745
No. of measured, independent and observed [*I* > 2σ(*I*)] reflections	15383, 3447, 2821
*R*_int_	0.024
(sin θ/λ)_max_ (Å^−1^)	0.626

Refinement	
*R*[*F* ^2^ > 2σ(*F* ^2^)], *wR*(*F* ^2^), *S*	0.037, 0.101, 1.03
No. of reflections	3447
No. of parameters	201
No. of restraints	1
H-atom treatment	H-atom parameters constrained
Δρ_max_, Δρ_min_ (e Å^−3^)	0.22, −0.13
Absolute structure	Flack *x* determined using 1136 quotients [(*I* ^+^)−(*I* ^−^)]/[(*I* ^+^)+(*I* ^−^)] (Parsons *et al.*, 2013 ▸)
Absolute structure parameter	0.0 (5)

Computer programs: *APEX2* and *SAINT* (Bruker, 2016 ▸), *SHELXS97* and *SHELXL97* (Sheldrick, 2008 ▸), *SHELXL2018/3* (Sheldrick, 2015 ▸), *ORTEP-3 for Windows* (Farrugia, 2012 ▸) and *PLATON* (Spek, 2020 ▸).

## supplementary crystallographic information

### Crystal data

**Table:**

C_20_H_23_NO	*F*(000) = 316
*M**_r_* = 293.39	*D*_x_ = 1.153 Mg m^−^^3^
Monoclinic, *P*2_1_	Mo *K*α radiation, λ = 0.71073 Å
*a* = 8.3063 (4) Å	Cell parameters from 2821 reflections
*b* = 7.5842 (4) Å	θ = 2.6–26.4°
*c* = 13.8410 (7) Å	µ = 0.07 mm^−^^1^
β = 104.174 (2)°	*T* = 296 K
*V* = 845.39 (7) Å^3^	Block, white
*Z* = 2	0.30 × 0.25 × 0.25 mm

### Data collection

**Table:**

Bruker SMART APEXII CCD diffractometer	2821 reflections with *I* > 2σ(*I*)
Radiation source: fine-focus sealed tube	*R*_int_ = 0.024
ω and φ scans	θ_max_ = 26.4°, θ_min_ = 2.6°
Absorption correction: multi-scan (SADABS; Bruker, 2016)	*h* = −10→9
*T*_min_ = 0.697, *T*_max_ = 0.745	*k* = −9→9
15383 measured reflections	*l* = −17→17
3447 independent reflections	

### Refinement

**Table:**

Refinement on *F*^2^	Hydrogen site location: inferred from neighbouring sites
Least-squares matrix: full	H-atom parameters constrained
*R*[*F*^2^ > 2σ(*F*^2^)] = 0.037	*w* = 1/[σ^2^(*F*_o_^2^) + (0.0534*P*)^2^ + 0.0626*P*] where *P* = (*F*_o_^2^ + 2*F*_c_^2^)/3
*wR*(*F*^2^) = 0.101	(Δ/σ)_max_ < 0.001
*S* = 1.03	Δρ_max_ = 0.22 e Å^−^^3^
3447 reflections	Δρ_min_ = −0.13 e Å^−^^3^
201 parameters	Absolute structure: Flack *x* determined using 1136 quotients [(*I*^+^)-(*I*^-^)]/[(*I*^+^)+(*I*^-^)] (Parsons *et al.*, 2013)
1 restraint	Absolute structure parameter: 0.0 (5)
Primary atom site location: difference Fourier map	

### Special details

**Table:**

Geometry. All esds (except the esd in the dihedral angle between two l.s. planes) are estimated using the full covariance matrix. The cell esds are taken into account individually in the estimation of esds in distances, angles and torsion angles; correlations between esds in cell parameters are only used when they are defined by crystal symmetry. An approximate (isotropic) treatment of cell esds is used for estimating esds involving l.s. planes.

### Fractional atomic coordinates and isotropic or equivalent isotropic displacement parameters (Å^2^)

**Table:**

	*x*	*y*	*z*	*U*_iso_*/*U*_eq_	
C2	0.3371 (3)	0.2057 (3)	0.31568 (16)	0.0457 (5)	
H2	0.377220	0.270737	0.378151	0.055*	
C3	0.1580 (3)	0.2626 (4)	0.2699 (2)	0.0565 (6)	
H3A	0.155366	0.388579	0.257504	0.068*	
H3B	0.117951	0.203547	0.206358	0.068*	
C4	0.0435 (3)	0.2199 (4)	0.3367 (2)	0.0679 (7)	
H4A	−0.070712	0.231457	0.298638	0.081*	
H4B	0.061680	0.304992	0.390657	0.081*	
C5	0.0700 (3)	0.0344 (4)	0.38074 (17)	0.0567 (6)	
H5	−0.038621	−0.023553	0.368498	0.068*	
C6	0.1821 (3)	−0.0774 (3)	0.33117 (17)	0.0481 (5)	
H6	0.209751	−0.182736	0.372930	0.058*	
C7	0.1060 (3)	−0.1457 (3)	0.22655 (17)	0.0502 (5)	
C8	−0.0600 (3)	−0.1247 (4)	0.1781 (2)	0.0659 (7)	
H8	−0.129032	−0.058009	0.207568	0.079*	
C9	−0.1240 (5)	−0.2032 (5)	0.0855 (2)	0.0812 (10)	
H9	−0.235412	−0.188306	0.053477	0.097*	
C10	−0.0235 (6)	−0.3020 (5)	0.0415 (2)	0.0890 (11)	
H10	−0.066947	−0.354500	−0.020068	0.107*	
C11	0.1404 (5)	−0.3236 (4)	0.0881 (2)	0.0824 (10)	
H11	0.208456	−0.390605	0.058133	0.099*	
C12	0.2053 (4)	−0.2460 (4)	0.1796 (2)	0.0637 (7)	
H12	0.317158	−0.260974	0.210498	0.076*	
C13	0.4446 (3)	0.2557 (3)	0.24606 (16)	0.0471 (5)	
C14	0.4544 (3)	0.1539 (4)	0.16487 (19)	0.0584 (6)	
H14	0.395691	0.048462	0.152879	0.070*	
C15	0.5502 (4)	0.2063 (5)	0.1012 (2)	0.0715 (8)	
H15	0.555322	0.136101	0.047006	0.086*	
C16	0.6372 (4)	0.3607 (5)	0.1175 (2)	0.0783 (9)	
H16	0.701865	0.395466	0.074701	0.094*	
C17	0.6289 (4)	0.4638 (4)	0.1971 (3)	0.0775 (9)	
H17	0.688101	0.568970	0.208368	0.093*	
C18	0.5326 (3)	0.4126 (4)	0.2611 (2)	0.0632 (7)	
H18	0.526974	0.484386	0.314655	0.076*	
C19	0.4825 (3)	−0.0674 (3)	0.39016 (16)	0.0476 (5)	
C20	0.6396 (3)	0.0380 (4)	0.41982 (19)	0.0617 (7)	
H20A	0.724197	−0.031394	0.462980	0.093*	
H20B	0.619811	0.142671	0.454141	0.093*	
H20C	0.675456	0.070131	0.361322	0.093*	
C21	0.1416 (4)	0.0413 (5)	0.4921 (2)	0.0733 (8)	
H21A	0.155370	−0.076398	0.518301	0.110*	
H21B	0.067488	0.105297	0.522752	0.110*	
H21C	0.247396	0.099569	0.506051	0.110*	
N1	0.3411 (2)	0.0152 (2)	0.33954 (13)	0.0433 (4)	
O1	0.4827 (2)	−0.2245 (3)	0.41220 (16)	0.0707 (5)	

### Atomic displacement parameters (Å^2^)

**Table:**

	*U*^11^	*U*^22^	*U*^33^	*U*^12^	*U*^13^	*U*^23^
C2	0.0422 (11)	0.0485 (13)	0.0457 (11)	0.0023 (10)	0.0097 (9)	−0.0057 (10)
C3	0.0478 (13)	0.0552 (14)	0.0642 (14)	0.0106 (11)	0.0097 (11)	0.0003 (12)
C4	0.0484 (14)	0.0814 (19)	0.0771 (17)	0.0154 (14)	0.0216 (13)	−0.0054 (15)
C5	0.0388 (12)	0.0813 (18)	0.0536 (13)	−0.0051 (12)	0.0180 (10)	−0.0059 (13)
C6	0.0379 (11)	0.0585 (13)	0.0484 (11)	−0.0038 (10)	0.0117 (9)	0.0029 (11)
C7	0.0493 (13)	0.0504 (12)	0.0509 (12)	−0.0083 (11)	0.0123 (10)	0.0014 (11)
C8	0.0556 (15)	0.0738 (19)	0.0623 (15)	−0.0092 (14)	0.0029 (12)	−0.0028 (14)
C9	0.080 (2)	0.085 (2)	0.0654 (17)	−0.0241 (17)	−0.0076 (16)	0.0078 (16)
C10	0.122 (3)	0.086 (2)	0.0541 (16)	−0.035 (2)	0.0132 (19)	−0.0097 (16)
C11	0.109 (3)	0.071 (2)	0.0726 (19)	−0.0196 (18)	0.0327 (19)	−0.0186 (16)
C12	0.0700 (17)	0.0594 (15)	0.0644 (15)	−0.0064 (13)	0.0217 (13)	−0.0078 (13)
C13	0.0420 (12)	0.0468 (12)	0.0502 (12)	0.0015 (10)	0.0068 (9)	0.0034 (10)
C14	0.0599 (14)	0.0624 (15)	0.0547 (14)	−0.0057 (12)	0.0173 (12)	−0.0007 (11)
C15	0.0708 (17)	0.088 (2)	0.0616 (15)	0.0017 (16)	0.0277 (14)	0.0080 (15)
C16	0.0658 (18)	0.092 (2)	0.083 (2)	−0.0003 (17)	0.0287 (15)	0.0310 (19)
C17	0.0677 (17)	0.0639 (18)	0.102 (2)	−0.0122 (14)	0.0221 (17)	0.0219 (17)
C18	0.0586 (15)	0.0526 (14)	0.0755 (16)	−0.0018 (12)	0.0110 (13)	0.0047 (13)
C19	0.0417 (12)	0.0580 (14)	0.0444 (11)	0.0082 (11)	0.0129 (9)	0.0067 (10)
C20	0.0361 (11)	0.0821 (18)	0.0650 (14)	0.0069 (12)	0.0085 (10)	0.0051 (14)
C21	0.0709 (18)	0.091 (2)	0.0638 (15)	−0.0030 (16)	0.0278 (13)	0.0006 (16)
N1	0.0335 (9)	0.0520 (10)	0.0445 (9)	0.0014 (8)	0.0100 (7)	0.0023 (8)
O1	0.0595 (12)	0.0657 (12)	0.0843 (13)	0.0117 (9)	0.0125 (10)	0.0206 (10)

### Geometric parameters (Å, º)

**Table:**

C2—N1	1.481 (3)	C11—C12	1.381 (4)
C2—C13	1.513 (3)	C11—H11	0.9300
C2—C3	1.530 (3)	C12—H12	0.9300
C2—H2	0.9800	C13—C14	1.382 (4)
C3—C4	1.515 (4)	C13—C18	1.386 (4)
C3—H3A	0.9700	C14—C15	1.382 (4)
C3—H3B	0.9700	C14—H14	0.9300
C4—C5	1.528 (4)	C15—C16	1.366 (5)
C4—H4A	0.9700	C15—H15	0.9300
C4—H4B	0.9700	C16—C17	1.366 (5)
C5—C21	1.511 (4)	C16—H16	0.9300
C5—C6	1.539 (3)	C17—C18	1.386 (4)
C5—H5	0.9800	C17—H17	0.9300
C6—N1	1.475 (3)	C18—H18	0.9300
C6—C7	1.522 (3)	C19—O1	1.230 (3)
C6—H6	0.9800	C19—N1	1.364 (3)
C7—C8	1.387 (3)	C19—C20	1.499 (4)
C7—C12	1.394 (4)	C20—H20A	0.9600
C8—C9	1.395 (4)	C20—H20B	0.9600
C8—H8	0.9300	C20—H20C	0.9600
C9—C10	1.371 (6)	C21—H21A	0.9600
C9—H9	0.9300	C21—H21B	0.9600
C10—C11	1.366 (5)	C21—H21C	0.9600
C10—H10	0.9300		
			
N1—C2—C13	113.69 (18)	C10—C11—C12	120.1 (3)
N1—C2—C3	109.49 (19)	C10—C11—H11	119.9
C13—C2—C3	109.34 (19)	C12—C11—H11	119.9
N1—C2—H2	108.1	C11—C12—C7	121.2 (3)
C13—C2—H2	108.1	C11—C12—H12	119.4
C3—C2—H2	108.1	C7—C12—H12	119.4
C4—C3—C2	112.3 (2)	C14—C13—C18	117.9 (2)
C4—C3—H3A	109.2	C14—C13—C2	122.6 (2)
C2—C3—H3A	109.2	C18—C13—C2	119.4 (2)
C4—C3—H3B	109.2	C15—C14—C13	121.0 (3)
C2—C3—H3B	109.2	C15—C14—H14	119.5
H3A—C3—H3B	107.9	C13—C14—H14	119.5
C3—C4—C5	112.9 (2)	C16—C15—C14	120.4 (3)
C3—C4—H4A	109.0	C16—C15—H15	119.8
C5—C4—H4A	109.0	C14—C15—H15	119.8
C3—C4—H4B	109.0	C17—C16—C15	119.6 (3)
C5—C4—H4B	109.0	C17—C16—H16	120.2
H4A—C4—H4B	107.8	C15—C16—H16	120.2
C21—C5—C4	110.9 (2)	C16—C17—C18	120.4 (3)
C21—C5—C6	110.1 (2)	C16—C17—H17	119.8
C4—C5—C6	111.90 (19)	C18—C17—H17	119.8
C21—C5—H5	107.9	C13—C18—C17	120.7 (3)
C4—C5—H5	107.9	C13—C18—H18	119.7
C6—C5—H5	107.9	C17—C18—H18	119.7
N1—C6—C7	113.10 (17)	O1—C19—N1	121.4 (2)
N1—C6—C5	109.31 (19)	O1—C19—C20	120.0 (2)
C7—C6—C5	117.12 (19)	N1—C19—C20	118.5 (2)
N1—C6—H6	105.4	C19—C20—H20A	109.5
C7—C6—H6	105.4	C19—C20—H20B	109.5
C5—C6—H6	105.4	H20A—C20—H20B	109.5
C8—C7—C12	118.0 (2)	C19—C20—H20C	109.5
C8—C7—C6	123.5 (2)	H20A—C20—H20C	109.5
C12—C7—C6	118.3 (2)	H20B—C20—H20C	109.5
C7—C8—C9	120.3 (3)	C5—C21—H21A	109.5
C7—C8—H8	119.8	C5—C21—H21B	109.5
C9—C8—H8	119.8	H21A—C21—H21B	109.5
C10—C9—C8	120.3 (3)	C5—C21—H21C	109.5
C10—C9—H9	119.8	H21A—C21—H21C	109.5
C8—C9—H9	119.8	H21B—C21—H21C	109.5
C11—C10—C9	120.1 (3)	C19—N1—C6	117.61 (18)
C11—C10—H10	120.0	C19—N1—C2	122.16 (19)
C9—C10—H10	120.0	C6—N1—C2	118.46 (17)
			
N1—C2—C3—C4	56.0 (3)	N1—C2—C13—C18	−141.6 (2)
C13—C2—C3—C4	−178.8 (2)	C3—C2—C13—C18	95.7 (3)
C2—C3—C4—C5	−45.0 (3)	C18—C13—C14—C15	0.6 (4)
C3—C4—C5—C21	112.4 (3)	C2—C13—C14—C15	178.1 (2)
C3—C4—C5—C6	−11.0 (3)	C13—C14—C15—C16	0.0 (5)
C21—C5—C6—N1	−68.0 (3)	C14—C15—C16—C17	−0.2 (5)
C4—C5—C6—N1	55.8 (3)	C15—C16—C17—C18	0.0 (5)
C21—C5—C6—C7	161.7 (2)	C14—C13—C18—C17	−0.8 (4)
C4—C5—C6—C7	−74.5 (3)	C2—C13—C18—C17	−178.4 (2)
N1—C6—C7—C8	−133.9 (2)	C16—C17—C18—C13	0.6 (4)
C5—C6—C7—C8	−5.4 (4)	O1—C19—N1—C6	13.1 (3)
N1—C6—C7—C12	51.0 (3)	C20—C19—N1—C6	−166.3 (2)
C5—C6—C7—C12	179.5 (2)	O1—C19—N1—C2	177.7 (2)
C12—C7—C8—C9	0.3 (4)	C20—C19—N1—C2	−1.7 (3)
C6—C7—C8—C9	−174.8 (3)	C7—C6—N1—C19	−108.2 (2)
C7—C8—C9—C10	0.1 (5)	C5—C6—N1—C19	119.4 (2)
C8—C9—C10—C11	−0.3 (5)	C7—C6—N1—C2	86.6 (2)
C9—C10—C11—C12	0.1 (5)	C5—C6—N1—C2	−45.8 (2)
C10—C11—C12—C7	0.4 (5)	C13—C2—N1—C19	64.0 (3)
C8—C7—C12—C11	−0.6 (4)	C3—C2—N1—C19	−173.40 (18)
C6—C7—C12—C11	174.8 (2)	C13—C2—N1—C6	−131.6 (2)
N1—C2—C13—C14	40.9 (3)	C3—C2—N1—C6	−8.9 (3)
C3—C2—C13—C14	−81.8 (3)		

### Hydrogen-bond geometry (Å, º)

**Table:**

*D*—H···*A*	*D*—H	H···*A*	*D*···*A*	*D*—H···*A*
C20—H20*B*···O1^i^	0.96	2.44	3.292 (3)	148

Symmetry code: (i) −*x*+1, *y*+1/2, −*z*+1.
